# Adults with Attention Deficit Hyperactivity Disorder Report High Symptom Levels of Troubled Sleep, Restless Legs, and Cataplexy

**DOI:** 10.3389/fpsyg.2017.01621

**Published:** 2017-09-20

**Authors:** Bjørn Bjorvatn, Erlend J. Brevik, Astri J. Lundervold, Anne Halmøy, Maj-Britt Posserud, Johanne T. Instanes, Jan Haavik

**Affiliations:** ^1^Department of Global Public Health and Primary Care, University of Bergen Bergen, Norway; ^2^Norwegian Competence Center for Sleep Disorders, Haukeland University Hospital Bergen, Norway; ^3^Division of Psychiatry, Haukeland University Hospital Bergen, Norway; ^4^Department of Biomedicine, K.G. Jebsen Centre for Neuropsychiatric Disorders, University of Bergen Bergen, Norway; ^5^Department of Biological and Medical Psychology, University of Bergen Bergen, Norway

**Keywords:** sleep, sleepiness, snoring, apnea, restless legs, ADHD, stimulant medication, subtypes

## Abstract

**Objective:** To compare the occurrence of a spectrum of different self-reported sleep problems in adults with ADHD and a control group, and to study the impact of current ADHD medication use and clinical ADHD subtype.

**Method:** Cross-sectional study of 268 clinically ascertained adult ADHD patients (DSM-IV criteria) and 202 randomly selected controls. Sleep problems were self-reported using validated questions, partly from Global Sleep Assessment Questionnaire.

**Results:** ADHD patients reported more sleep problems than controls: Lifetime occurrence of sleep problems (82.6 vs. 36.5%), hypnotics use (61.4 vs. 20.2%), current sleep duration below 6 h (26.6 vs. 7.6%), and symptoms/signs during the past 4 weeks of excessive daytime sleepiness, cataplexy, loud snoring, breathing pauses during sleep, restless legs, and periodic limb movements in sleep (significant odds ratios ranged from 1.82 to 14.55). Current ADHD medication use was associated with less cataplexy compared with not using medication. Patients with inattentive subtype reported better sleep quality and less restless legs than patients with hyperactive/impulsive subtypes.

**Conclusions:** Adults with ADHD reported a very high occurrence of many different self-reported sleep problems, underlining the importance of screening for sleep disorders. Among the ADHD patients, medication use was not associated with more sleep-related symptoms, but in fact less cataplexy. When comparing ADHD subtypes, the inattentive subtype was associated with less sleep problems.

## Introduction

Attention-deficit/hyperactivity disorder (ADHD) is a common neurodevelopmental disorder. The main symptoms are inattention, hyperactivity, and impulsivity. Based on these symptoms the disorder can be categorized into three subtypes/presentations: an inattentive subtype (IA), a hyperactive/impulsive subtype (HI), and a combined subtype in which both inattentive and hyperactive/impulsive symptoms are present (American Psychiatric Association, 2000). Patients with ADHD also have many other symptoms, including sleep problems, which are reported to occur in 25–55% of patients with ADHD (Hvolby, 2015). In a recent systematic literature review, sleep problems were reported to be among the most common co-morbidities associated with ADHD (Instanes et al., 2016).

Poor sleep often leads to inattention/lack of concentration and mood swings, symptoms also typically seen in ADHD. Thus, it has been suggested that some patients may have been misdiagnosed with ADHD instead of a primary sleep disorder (Philipsen et al., 2006; Yoon et al., 2012; Bioulac et al., 2015). Alternatively, sleep problems may be considered an intrinsic feature of ADHD (Yoon et al., 2012; Hvolby, 2015; Hysing et al., 2016). Moreover, some patients may have co-morbid ADHD and sleep disorder (Bioulac et al., 2015). Overall, the distinction between having ADHD with sleep problems, having a sleep disorder with ADHD-like symptoms, or having co-morbid ADHD and sleep disorder is blurred and needs more systematic exploration (Oosterloo et al., 2006; Yoon et al., 2012; Bioulac et al., 2015; Hvolby, 2015).

Most studies investigating sleep problems among ADHD patients have been performed in children/adolescents (Philipsen et al., 2006; Yoon et al., 2012; Bioulac et al., 2015; Hvolby, 2015). However, it has been proposed that sleep-related problems are even more common in adults than in children with ADHD, and that the type of sleep problems may depend on age (Surman et al., 2009; Yoon et al., 2012; Hvolby, 2015).

The effects of ADHD medication on sleep are controversial (Surman and Roth, 2011; Yoon et al., 2012; Hvolby, 2015; Instanes et al., 2016). Both stimulant medication and atomoxetine have been reported to cause sleep problems as an adverse effect (Adler et al., 2009; Kirov and Brand, 2014), while other studies show that stimulants may improve sleep quality and sleep efficiency (Kooij et al., 2001; Boonstra et al., 2007; Sobanski et al., 2008). Again, most studies have been performed among children/adolescents with ADHD, and more research on the effects of ADHD medication on sleep in adults is warranted.

The relation between sleep problems and ADHD subtypes is another open question. Some studies suggest that sleep quality (Yoon et al., 2013) and daytime sleepiness (LeBourgeois et al., 2004; Chiang et al., 2010) are worse in ADHD patients with the inattentive subtype, whereas other studies suggest that sleep problems are worse in ADHD subtypes with hyperactivity/impulsivity (Corkum et al., 1999; Mayes et al., 2009; Silvestri et al., 2009). The ADHD combined subtype is characterized by an overall higher symptom severity compared with the other subtypes (Wilens et al., 2009), which may also influence the occurrence of sleep problems.

Research on the association between ADHD and sleep problems has not focused on sleep duration (Yoon et al., 2012; Hvolby, 2015; Instanes et al., 2016). However, the negative impact on health of short sleep duration, usually defined as <6 h of sleep, has received increased focus (Tobaldini et al., 2017). There is evidence linking short sleep duration with increased risk of obesity, diabetes, heart disease, and mortality (Liu et al., 2017; Tobaldini et al., 2017). Thus, it is of interest to study sleep duration in ADHD patients, and the impact of ADHD medication and ADHD subtypes on sleep duration.

Sleep problems can be investigated in many different ways. Some sleep disorders require objective measurements for proper diagnosis, e.g., sleep apnea, whereas other disorders are purely based on subjective reports from the patients, e.g., restless legs syndrome (American Academy of Sleep Medicine, 2014). Most studies use self-report questionnaires. However, the quality and validity of these questionnaires differ substantially. In a recent clinical review (Klingman et al., 2017), the authors identified and evaluated different screening questionnaires for sleep disorders, and concluded that the Global Sleep Assessment Questionnaire (GSAQ) was the most suitable as a general sleep disorders screener, assessing many different sleep disorders (Roth et al., 2002).

The first aim of the present study was to compare the occurrence of different types of self-reported sleep problems, using selected and validated questions, between a large sample of adults with clinically ascertained ADHD and a randomly selected control group. We hypothesized that the ADHD patients would report more sleep problems than the controls. The second aim was to compare the occurrence of sleep problems in adult ADHD patients currently using and not using ADHD medication. The third aim was to compare the occurrence of these sleep problems in different subtypes of ADHD (IA vs. subtypes with HI).

## Materials and methods

### Sample

This cross-sectional study is part of an ongoing project on adults with ADHD in Norway (http://www.uib.no/kgj-npd). The sample comprised adults with ADHD (*n* = 268), clinically diagnosed by psychiatrists/psychologists according to the Diagnostic and Statistical Manual of Mental Disorders (*DSM-IV*) criteria (American Psychiatric Association, 2000). The first patients were recruited from regional expert committees on ADHD (*n* = 57), subsequent patients were recruited from clinical psychologists and psychiatrists in out-patient clinics nationwide (*n* = 211). The data were collected between 2011 and 2015. More details about the study can be found elsewhere (Brevik et al., 2017).

In this project we relied on detailed information provided by both participants and, for the ADHD patients, also from their treating physicians. During the course of the project, we tested two different versions of clinician-rated questionnaires; one extended version with detailed treatment details and a simplified version focusing on validation of the diagnoses (to save time for the participating clinicians). In the final sample, about half of the patients were recruited using each of these questionnaires, but there was no indication of group differences between the patients recruited by these slightly different protocols (data not shown). Thus, for half of the patients (*n* = 135/50.4%), we had access to clinician reported information on ADHD subtype and medication use. Of these, 54 patients belonged to the IA, 6 to the HI, and 75 to the combined subtype. Regarding medication use, 94 patients were currently using ADHD medication (69 patients on methylphenidate, 12 on amphetamines, 3 on atomoxetine, 7 on a combination, and 3 patients were on an unknown ADHD medication), whereas 36 patients were not using ADHD medication.

The controls (*n* = 202) were randomly selected and invited from the nation-wide Medical Birth Registry of Norway. To allow for co-morbidities and reduce selection bias, no formal exclusion criteria were used in either sample. All patients and controls were born in Norway; 90.2% of patients and 92.9% of controls stated that both parents also were of Norwegian ancestry (*p* = 0.4). All participants signed a written informed consent, and the study was approved by the Norwegian Regional Committee for Medical and Health Research Ethics, REC West [IRB #3 (FWA00009490, IRB00001872)].

### Sleep variables

The participants completed a comprehensive sleep questionnaire (see **Appendix**). The questions about different sleep disorders (except the question on cataplexy) were from the GSAQ (Roth et al., 2002). To ease interpretation and readability, most questions were dichotomized in the regression analyses as “never” vs. “sometimes”/“usually”/“always” (all logistic regression analyses) or “never”/“sometimes” vs. “usually”/“always” (only for the logistic regression analyses with ADHD as dependent variable).

### Statistics

Data were analyzed using SPSS v-23. Chi-square tests were used to compare categorical variables and independent samples *t*-test to compare continuous variables. Logistic regression analyses adjusted for sex and age, with ADHD as dependent variable, were used to investigate the association between different self-reported sleep problems and having ADHD. Similar adjusted logistic regression analyses were run on the clinical sample of ADHD patients (i.e., the subset of ADHD patients with detailed clinical data), with “not using ADHD medication” as the dependent variable, in order to investigate the association between “currently using” and “not using medication” on occurrence of sleep problems. Furthermore, adjusted logistic regression analyses were run on the clinical sample of ADHD patients with the inattentive ADHD subtype as the dependent variable, in order to investigate the association between the IA and the subtypes with HI (the HI and combined subtypes were combined due to few patients with the HI subtype) on occurrence of sleep problems. Missing values for the different sleep questions varied from 1.7 to 6.6% (breathing pauses during sleep). Only valid cases were used in the analyses. Significance levels were set at 0.05.

## Results

The sex distribution between controls and ADHD patients was similar (Table 1). Mean age was 36.5 and 38.1 years among controls and ADHD patients, respectively (*p* = 0.06). Among ADHD patients, 82.6% reported to have had sleep problems (in general) that lasted 1 month or longer, compared to 36.5% among the controls (Table 1). Having used hypnotics were reported by 61.4% compared to 20.2% in the ADHD and control groups, respectively. Self-reported sleep duration in the ADHD group was 6.5 h compared to 6.9 h among the controls [*t*_(451)_ = 3.2, *p* = 0.002], and 26.6% of the ADHD patients reported sleeping <6 h, compared to 7.6% of the controls (Table 1). ADHD patients more often reported to belong to the extremes of circadian type, with especially many patients reporting to be definite evening types compared to controls.

**Table 1:** Self-reported sleep problems in Norwegian adults with clinically ascertained ADHD (*n* = 268) compared to a representative control group (*n* = 202).

**Characteristics and self-reported sleep problems**	**% (*****n*****)**		**Chi-square (df)**	***p*-value^a^**
	**Controls**	**ADHD patients**		
Sex			0.4 (1)	0.55
Males	37.1 (75)	40.3 (108)		
Females	62.9 (127)	59.7 (160)		
Age			**27.7 (2)**	**<0.0001**
18–29 years	21.3 (43)	24.3 (65)		
30–44 years	67.8 (137)	46.6 (125)		
45+ years	10.9 (22)	29.1 (78)		
Ever had sleep problems?			**100.5 (1)**	**<0.0001**
Yes	36.5 (72)	82.6 (218)		
No	63.5 (125)	17.4 (46)		
Ever used hypnotics?			**76.2 (1)**	**<0.0001**
Yes	20.2 (40)	61.4 (162)		
No	79.8 (158)	38.6 (102)		
Sleep quality			**80.1 (4)**	**<0.0001**
Very good	32.3 (63)	10.1 (26)		
Good	47.7 (93)	30.6 (79)		
Neither good nor bad	13.3 (26)	28.7 (74)		
Pretty bad	6.2 (12)	25.2 (65)		
Very bad	0.5 (1)	5.4 (14)		
Sleep duration			**25.5 (1)**	**<0.0001**
Below 6 h	7.6 (15)	26.6 (68)		
6 h or more	92.4 (182)	73.4 (188)		
Circadian type			**20.7(4)**	**0.0004**
Definite morning type	7.6 (15)	10.7 (28)		
Moderate morning type	25.3 (50)	18.3 (48)		
Intermediate type	21.7 (43)	20.6 (54)		
Moderate evening type	33.8 (67)	23.7 (62)		
Definite evening type	11.6 (23)	26.7 (70)		
Excessive daytime sleepiness^b^			**17.7 (3)**	**0.0005**
Never	57.1 (113)	42.4 (112)		
Sometimes	39.9 (79)	45.1 (119)		
Usually	2.5 (5)	10.6 (28)		
Always	0.5 (1)	1.9 (5)		
Cataplexy^b^			**59.4 (3)**	**<0.0001**
Never	95.5 (189)	65.6 (172)		
Sometimes	4.0 (8)	28.6 (75)		
Usually	0.5 (1)	5.3 (14)		
Always	0.0 (0)	0.4 (1)		
Loud snoring^b^			**26.8 (3)**	**<0.0001**
Never	56.9 (111)	35.9 (92)		
Sometimes	33.3 (65)	40.6 (104)		
Usually	9.2 (18)	17.6 (45)		
Always	0.5 (1)	5.9 (15)		
Breathing pauses during sleep^b^			**36.3 (3)**	**<0.0001**
Never	92.9 (182)	70.2 (170)		
Sometimes	5.1 (10)	21.1 (51)		
Usually	2.0 (4)	5.4 (13)		
Always	0.0 (0)	3.3 (8)		
Restless legs^b^			**76.3 (3)**	**<0.0001**
Never	74.7 (148)	37.0 (97)		
Sometimes	23.2 (46)	39.3 (103)		
Usually	1.0 (2)	18.3 (48)		
Always	1.0 (2)	5.3 (14)		
Periodic limb movements^b^			**61.8 (3)**	**<0.0001**
Never	81.6 (160)	45.5 (115)		
Sometimes	15.3 (30)	41.9 (106)		
Usually	2.6 (5)	11.9 (30)		
Always	0.5 (1)	0.8 (2)		

a *Pearson Chi-square, with Yates' correction for continuity when used in a 2 × 2 table*.

b *Symptoms/signs experienced during the past 4 weeks*.

*df, degrees of freedom. Significant values are shown in **bold***.

**Figure 1:**
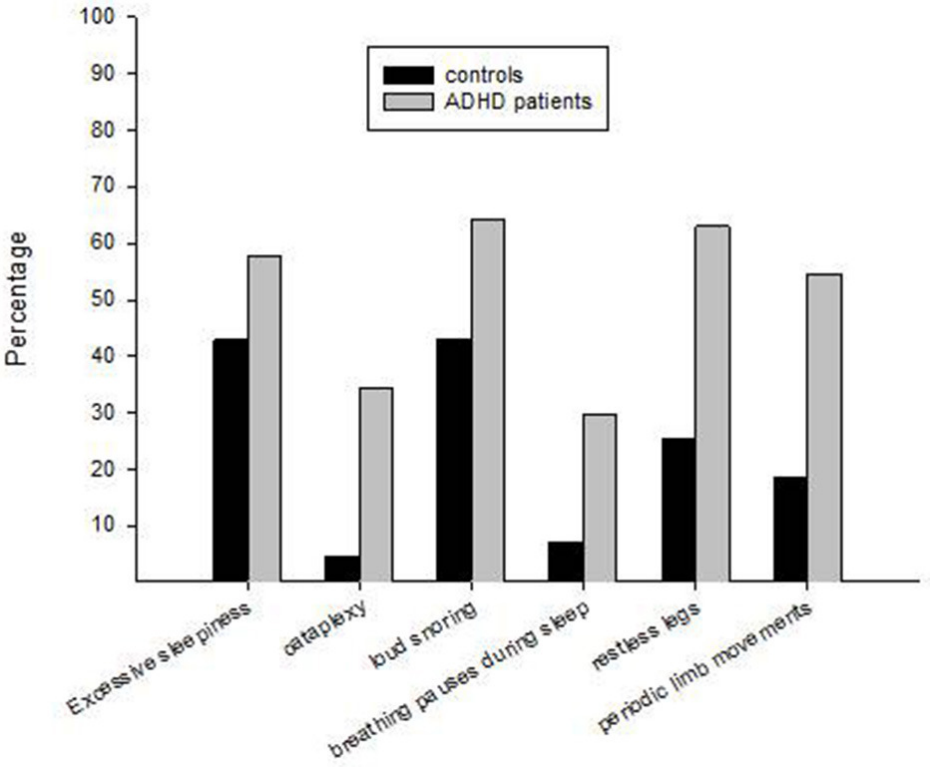
Self-reported symptoms/signs of specific sleep disorders in Norwegian adults with clinically ascertained ADHD (*n* = 268) compared to a representative control group (*n* = 202). The percentages indicate frequencies of symptoms/signs experienced “sometimes” or more often during the past 4 weeks.

ADHD patients reported more symptoms/signs of specific sleep disorders compared with controls. This was evident for excessive daytime sleepiness, cataplexy, loud snoring, breathing pauses during sleep, restless legs, and periodic limb movements in sleep (Table 1, Figure 1). When adjusting for sex and age in the logistic regression analyses, having ADHD was markedly associated with the sleep disorders (defined as reporting such disorders “sometimes,” “usually,” or “always”), ranging from almost a two-fold increase (excessive daytime sleepiness) to an 11.5-fold increase (cataplexy) compared to controls (Table 2). The association between ADHD and the self-reported sleep disorders were 2.5- to 3-fold stronger for excessive daytime sleepiness and restless legs when the sleep disorders were reported “usually” or “always” (Table 2).

**Table 2:** Logistic regression analyses with self-reported sleep variables as predictors and clinically ascertained ADHD among Norwegian adults as the dependent variable.

	**Adjusted OR^a^ (95% CI) *n* = 438–462**		**Adjusted OR^a^ (95% CI) *n* = 438–462**
Ever had sleep problems?			
No	1.00		
Yes	**8.90 (5.69–13.92)**		
Ever used hypnotics?			
No	1.00		
Yes	**6.47 (4.20–9.97)**		
Sleep quality			
Good-very good	1.00		
Intermediate-very bad	**6.07 (3.93–9.39)**		
Sleep duration			
6 h or more	1.00		
Below 6 h	**4.63 (2.54–8.42)**		
Excessive daytime sleepiness^b^			
Never	1.00	Never-sometimes	1.00
Sometimes-always	**1.82 (1.25–2.65)**	Usually-always	**4.65 (1.90–11.38)**
Cataplexy^b^			
Never	1.00	Never-sometimes	1.00
Sometimes-always	**11.53 (5.61–23.69)**	Usually-always	**12.19 (1.59–93.34)**
Loud snoring^b^			
Never	1.00	Never-sometimes	1.00
Sometimes-always	**2.31 (1.55–3.43)**	Usually-always	**2.74 (1.55–4.84)**
Breathing pauses during sleep^b^			
Never	1.00	Never-sometimes	1.00
Sometimes-always	**5.45 (2.92–10.19)**	Usually-always	**4.18 (1.40–12.50)**
Restless legs^b^			
Never	1.00	Never-sometimes	1.00
Sometimes-always	**4.95 (3.29–7.45)**	Usually-always	**14.55 (5.18–40.82)**
Periodic limb movements^b^			
Never	1.00	Never-sometimes	1.00
Sometimes-always	**5.30 (3.41–8.23)**	Usually-always	**4.47 (1.83–10.94)**

a *Adjusted regression analyses with sex and age as co-variates*.

b *Symptoms/signs experienced during the past 4 weeks*.

*Significant values are shown in **bold***.

Within the ADHD group, patients currently using ADHD medication reported better sleep quality, less cataplexy, and less restless legs compared to patients not using ADHD medication (Table 3). In the adjusted logistic regression analyses, cataplexy (OR = 2.47, CI = 1.10–5.53) remained significantly and positively associated with not using ADHD medication, whereas poor sleep quality (OR = 2.37, CI = 0.95–5.93) and restless legs (OR = 2.35, CI = 0.98–5.65) did not remain significantly associated with not using ADHD medication. None of the other sleep variables were significantly associated with ADHD medication status in the adjusted logistic regression analyses.

**Table 3:** Comparison of self-reported sleep problems in Norwegian adults with clinically ascertained ADHD diagnosis currently using (*n* = 94) or not using (*n* = 36) ADHD medication.

**Characteristics**	**% (*****n*****)**		**Chi-square (df)**	***p*-value^a^**
	**ADHD patients using medication**	**ADHD patients not using medication**		
Sex			<0.01 (1)	0.987
Males	34.0 (32)	36.1 (13)		
Females	66.0 (62)	63.9 (23)		
Age			2.6 (2)	0.270
18–29 years	36.2 (34)	22.2 (8)		
30–44 years	48.9 (46)	55.6 (20)		
45+ years	14.9 (14)	22.2 (8)		
Ever had sleep problems?			<0.01 (1)	1.000
Yes	85.9 (79)	86.1 (31)		
No	14.1 (13)	13.9 (5)		
Ever used hypnotics?			0.6 (1)	0.438
Yes	63.0 (58)	71.9 (23)		
No	37.0 (34)	28.1 (9)		
Sleep quality			**9.7 (4)**	**0.045**
Very good	13.5 (12)	8.6 (3)		
Good	28.1 (25)	14.3 (5)		
Neither good nor bad	31.5 (28)	28.6 (10)		
Pretty bad	22.5 (20)	28.6 (10)		
Very bad	4.5 (4)	20.0 (7)		
Sleep duration			1.8 (1)	0.183
Below 6 h	25.8 (23)	40.0 (14)		
6 h or more	74.2 (66)	60.0 (21)		
Circadian type			7.9 (4)	0.096
Definite morning type	3.3 (3)	16.7 (6)		
Moderate morning type	19.6 (18)	16.7 (6)		
Intermediate type	20.7 (19)	22.2 (8)		
Moderate evening type	22.8 (21)	22.2 (8)		
Definite evening type	33.7 (31)	22.2 (8)		
Excessive daytime sleepiness^b^			7.0 (3)	0.073
Never	47.8 (44)	30.6 (11)		
Sometimes	40.2 (37)	44.4 (16)		
Usually	9.8 (9)	25.0 (9)		
Always	2.2 (2)	0.0 (0)		
Cataplexy^b^			**9.3 (3)**	**0.026**
Never	70.3 (64)	50.0 (18)		
Sometimes	26.4 (24)	36.1 (13)		
Usually	2.2 (2)	13.9 (5)		
Always	1.1 (1)	0.0 (0)		
Loud snoring^b^			3.2 (3)	0.362
Never	40.2 (37)	32.4 (11)		
Sometimes	41.3 (38)	41.2 (14)		
Usually	16.3 (15)	17.6 (6)		
Always	2.2 (2)	8.8 (3)		
Breathing pauses during sleep^b^			0.1 (3)	0.992
Never	77.0 (67)	77.4 (24)		
Sometimes	17.2 (15)	16.1 (5)		
Usually	2.3 (2)	3.2 (1)		
Always	3.4 (3)	3.2 (1)		
Restless legs^b^			**13.0 (3)**	**0.005**
Never	44.4 (40)	25.0 (9)		
Sometimes	38.9 (35)	27.8 (10)		
Usually	14.4 (13)	38.9 (14)		
Always	2.2 (2)	8.3 (3)		
Periodic limb movements^b^			7.8 (3)	0.051
Never	45.6 (41)	54.3 (19)		
Sometimes	48.9 (44)	28.6 (10)		
Usually	5.6 (5)	14.3 (5)		
Always	0.0 (0)	2.9 (1)		

a *Pearson Chi-square, with Yates' correction for continuity when used in a 2 x 2 table*.

b *Symptoms/signs experienced during the past 4 weeks*.

*df, degrees of freedom. Significant values are shown in **bold***.

Within the ADHD group, there were few differences in the sleep variables between ADHD subtypes (combined+HI subtypes vs. IA subtype; Table 4). The exception was for restless legs, with fewer patients with the inattentive subtype reporting restless legs (Table 4). This finding was confirmed in the adjusted logistic regression analysis (OR = 0.29, CI = 0.13–0.63). Furthermore, sleep quality was negatively associated with the inattentive subtype (OR = 0.33, CI = 0.15–0.73), indicating that patients with the combined+HI subtypes reported poorer sleep quality than patients with the IA subtype. None of the other sleep variables were significantly associated with ADHD subtype in the adjusted logistic regression analyses.

**Table 4:** The influence of ADHD subtypes on self-reported sleep problems in Norwegian adults with clinically ascertained ADHD (*n* = 135).

**Characteristics**	**ADHD subtypes % (*****n*****)**		**Chi-square (df)**	***p*-value^a^**
	**Combined+HI**	**IA**		
Sex			2.5 (1)	0.115
Males	29.6 (24)	44.4 (24)		
Females	70.4 (57)	55.6 (30)		
Age			0.4 (2)	0.799
18–29 years	37.0 (30)	31.5 (17)		
30–44 years	48.1 (39)	51.9 (28)		
45+ years	14.8 (12)	16.7 (9)		
Ever had sleep problems?			<0.1 (1)	1.000
Yes	87.3 (69)	87.0 (47)		
No	12.7 (10)	13.0 (7)		
Ever used hypnotics?			<0.1 (1)	1.000
Yes	65.8 (52)	64.8 (35)		
No	34.2 (27)	35.2 (19)		
Sleep quality			8.8 (4)	0.066
Very good	6.6 (5)	15.1 (8)		
Good	22.4 (17)	35.8 (19)		
Neither good nor bad	31.6 (24)	22.6 (12)		
Pretty bad	25.0 (19)	22.6 (12)		
Very bad	14.5 (11)	3.8 (2)		
Sleep duration			1.8 (1)	0.182
Below 6 h	34.7 (26)	22.2 (12)		
6 h or more	65.3 (49)	77.8 (42)		
Circadian type			4.5 (4)	0.345
Definite morning type	6.4 (5)	3.7 (2)		
Moderate morning type	16.7 (13)	22.2 (12)		
Intermediate type	19.2 (15)	20.4 (11)		
Moderate evening type	19.2 (15)	29.6 (16)		
Definite evening type	38.5 (30)	24.1 (13)		
Excessive daytime sleepiness^b^			5.0 (3)	0.173
Never	38.0 (30)	44.4 (24)		
Sometimes	38.0 (30)	46.3 (25)		
Usually	21.5 (17)	7.4 (4)		
Always	2.5 (2)	1.9 (1)		
Cataplexy^b^			1.5 (2)	0.464
Never	59.5 (47)	69.8 (37)		
Sometimes	34.2 (27)	26.4 (14)		
Usually	6.3 (5)	3.8 (2)		
Always	0.0 (0)	0.0 (0)		
Loud snoring^b^			0.4 (3)	0.945
Never	42.1 (32)	39.6 (21)		
Sometimes	36.8 (28)	37.7 (20)		
Usually	15.8 (12)	18.9 (10)		
Always	5.3 (4)	3.8 (2)		
Breathing pauses during sleep^b^			0.6 (3)	0.904
Never	79.4 (54)	73.6 (39)		
Sometimes	14.7 (10)	18.9 (10)		
Usually	2.9 (2)	3.8 (2)		
Always	2.9 (2)	3.8 (2)		
Restless legs^b^			**9.3 (3)**	**0.025**
Never	28.2 (22)	53.7 (29)		
Sometimes	42.3 (33)	29.6 (16)		
Usually	23.1 (18)	14.8 (8)		
Always	6.4 (5)	1.9 (1)		
Periodic limb movements^b^			3.2 (3)	0.365
Never	46.7 (35)	55.6 (30)		
Sometimes	41.3 (31)	40.7 (22)		
Usually	10.7 (8)	3.7 (2)		
Always	1.3 (1)	0.0 (0)		

a *Pearson Chi-square, with Yates' correction for continuity when used in a 2 × 2 table*.

b *Symptoms/signs experienced during the past 4 weeks*.

*df, degrees of freedom. IA, Inattentive subtype. HI, Hyperactive/Impulsive subtype*.

*Significant values are shown in **bold***.

## Discussion

Our study demonstrated that a variety of specific self-reported sleep-related problems, including restless legs and cataplexy, were very common among adults with clinically ascertained ADHD. All the examined self-reported sleep variables were significantly associated with having ADHD, even when including age and sex as co-variates. Within the ADHD group, current use of ADHD medication was associated with less cataplexy. Furthermore, patients with the inattentive subtype reported less restless legs and better sleep quality compared with patients with the combined or hyperactive subtype.

There was a clear difference between ADHD patients and controls for reported sleep problems overall, with 82.6% of ADHD patients compared to 36.5% of controls having experienced a sleep problem lasting more than 1 month. In line with this, as many as 61.5% of ADHD patients reported having used hypnotics, which was three times more often than among controls. We recently published data on insomnia in this sample, showing that 66.8% of the ADHD patients compared with 28.8% of the controls fulfilled the DSM-IV criteria for insomnia disorder (Brevik et al., 2017). These findings clearly indicate that sleep problems are common and clinically severe among adults with ADHD, and corroborate findings from other studies (Surman et al., 2009; Fisher et al., 2014; Hvolby, 2015; Instanes et al., 2016).

To explore the association between ADHD and more specific sleep disorders, we investigated self-reported symptoms and signs of such sleep disorders during the past 4 weeks. Excessive daytime sleepiness and cataplexy are symptoms typically seen in hypersomnias (American Academy of Sleep Medicine, 2014). While excessive daytime sleepiness may also be present in other sleep disorders (e.g., sleep-related breathing disorders) and in psychiatric (e.g., depression) and somatic (e.g., hypothyroidism) disorders, cataplexy is considered a specific sign of narcolepsy type 1 (American Academy of Sleep Medicine, 2014). Narcolepsy is characterized by excessive daytime sleepiness, sleep attacks, REM sleep pathology and cataplexy (American Academy of Sleep Medicine, 2014). Both self-reported excessive daytime sleepiness and cataplexy were highly associated with ADHD in the present study, where strikingly, having ADHD was associated with a 12-fold increased risk of reporting cataplexy during the past 4 weeks. Other studies have also reported high associations between ADHD and hypersomnias (Oosterloo et al., 2006; Surman et al., 2009; Ohayon, 2013; Yoon et al., 2013; Bioulac et al., 2015). Both disorders are treated with stimulant medication, suggesting a possible relation between the two conditions (Instanes et al., 2016).

Reporting symptoms or signs of restless legs and periodic limb movements in sleep (American Academy of Sleep Medicine, 2014) were also strongly associated with having ADHD in the present study. Having ADHD was associated with a 14.5-fold increased risk of reporting restless legs “usually” or “always” during the past 4 weeks. Other studies with smaller samples also show a strong association between ADHD and restless legs/periodic limb movements in sleep (Wagner et al., 2004; Philipsen et al., 2005; Schredl et al., 2007; Zak et al., 2009). Whether some of the ADHD patients may be misdiagnosed or have restless legs syndrome as a co-morbid disorder is not possible to determine based on our findings.

Loud snoring and breathing pauses during sleep are symptoms/signs of obstructive sleep apnea (American Academy of Sleep Medicine, 2014). Having ADHD was associated with a 2- to 3-fold higher risk of self-reported loud snoring and a 4- to 6-fold higher risk of self-reported breathing pauses during sleep, dependent on the cut-off applied for having such complaints. Other studies have also found a high association between ADHD and sleep apnea (Ball et al., 1999; Surman et al., 2006; Levy et al., 2009; Youssef et al., 2011). Since both disorders may cause similar daytime symptoms, some authors have suggested that sleep apnea may be misdiagnosed as ADHD (Ball et al., 1999; Naseem et al., 2001; Bioulac et al., 2015). However, as sleep apnea can only be diagnosed by objective measures (American Academy of Sleep Medicine, 2014), we do not know whether some of our ADHD patients in fact suffer from this sleep disorder.

In addition to the strong association between ADHD and proxies for specific sleep disorders, adults with ADHD reported worse sleep quality and shorter sleep duration. About 27% of the patients reported <6 h of sleep compared to 7.6% among the controls. Considering the extensive research showing that short sleep duration is linked to somatic diseases (Liu et al., 2017; Tobaldini et al., 2017), this has profound implications. Studies show that adults with ADHD have a delayed circadian rhythm (Van Veen et al., 2010; Coogan and McGowan, 2017). We did not ask specific questions about circadian rhythm sleep-wake disorders, but included a question about circadian rhythm preference. In line with other studies (Van Veen et al., 2010), more adults with ADHD in the present study reported being definite evening types (26.7 vs. 11.6%). Surprisingly, more adults with ADHD also reported being definite morning types (10.7 vs. 7.6%). By this, our data suggest that ADHD patients more often belong to the extremes of circadian type. This finding warrants further investigation.

From a clinical perspective, our findings raise concern. For instance, as many as 8.7% of the patients reported breathing pauses during sleep “usually” or “always” during the past 4 weeks. It is strongly recommended to further investigate such complaints, since breathing pauses during sleep are indicative of sleep apnea (American Academy of Sleep Medicine, 2014). Our study cannot answer whether patients or controls who reported severe sleep problems have received sleep diagnostics. However, in clinical practice symptoms like sleep problems, daytime sleepiness, and concentration problems may easily be attributed to the ADHD condition as such, and not to other potentially serious disorders. Our study therefore stresses the importance of a thorough investigation of sleep in patients with ADHD, as co-morbid sleep disorders seem to be highly prevalent.

Within the ADHD group, patients not using ADHD medication reported more cataplexy, worse sleep quality, and more restless legs. Notably, the latter two symptoms did not reach significance in the logistic regressions with adjustment for age and sex. In line with the present findings, our recent paper on insomnia in this sample showed that patients using ADHD medication reported less insomnia symptoms (Brevik et al., 2017). Thus, our data suggest that ADHD medication may improve certain sleep-related symptoms in adults with ADHD. These findings are in line with several other studies (Kooij et al., 2001; Boonstra et al., 2007; Sobanski et al., 2008; Surman and Roth, 2011), but in contrast to studies showing that such medication may impair sleep (Adler et al., 2009; Kirov and Brand, 2014).

With regards to ADHD subtypes, patients with the inattentive subtype reported less restless legs and better sleep quality compared to the combined+hyperactive/impulsive subtype. These findings are in contrast to studies showing that sleep quality is worse (Yoon et al., 2013) and daytime sleepiness is higher (Oosterloo et al., 2006; Chiang et al., 2010) in the inattentive subtype. However, the study by Yoon et al. (2013) used Pittsburgh Sleep Quality Index (PSQI) as the measure of sleep quality. PSQI is a nine-item questionnaire that also includes questions not directly related to self-reported quality of sleep, possibly explaining the differences between our and their study. Similar to our study, one study showed that non-medicated adults with the inattentive subtype report better sleep quality than adults with the hyperactive/impulsive subtype (Mahajan et al., 2010). Furthermore, in line with our study, several other studies suggest that sleep problems are worse in the HI subtype (Corkum et al., 1999; Mayes et al., 2009; Silvestri et al., 2009; Van Veen et al., 2010). It is however noteworthy that most sleep variables did not differ depending on ADHD subtype. This may imply that the ADHD subtype does not have a major defining role in the type of sleep problem experienced.

The present study had several strengths and limitations. The large sample with clinically ascertained adult ADHD diagnosis was an asset. Similarly, the controls were representative of the Norwegian adult population. In a representative sample of 5,000 Norwegian adults, 40.5% reported to have ever experienced a sleep problem lasting more than 1 month, and 19% reported to have used hypnotics (Omvik et al., 2010), similar occurrences as reported among the controls in our study (36.5 and 20.2%, respectively). This confirms that the controls recruited were representative of the general Norwegian adult population. The age and sex distributions in patients and controls were rather similar, but still, we adjusted for these variables in the logistic regression analyses. Thus, age- or sex-dependent impact on the sleep variables were controlled for. Most questions about sleep problems came from validated questionnaires or from earlier published studies, strengthening the interpretation of the results and making comparisons to other studies possible. GSAQ is recommended as the questionnaire of choice when screening for sleep disorders (Klingman et al., 2017). The questionnaire is brief and covers several different sleep-related complaints, making it especially useful in clinical practice. However, it is not a diagnostic tool since only one question per sleep disorder is included. As all sleep data were self-reported, no formal sleep disorder diagnosis can be made. Our study included questions which may be difficult for participants to respond to, e.g., loud snoring, breathing pauses during sleep and periodic limb movements. These questions rely on reports from bed partners, introducing possible response bias. Furthermore, cataplexy was much more commonly reported than expected, and we recommend caution when interpreting the frequency of this complaint. Some participants may have misinterpreted the question, but even so, the large difference between controls and patients seems valid, as all participants responded to the same wording. Multiple statistical tests were performed, without controlling for the number of tests. Thus, careful interpretation of the findings is recommended, especially when the statistical analyses showed *p*-values close to 0.05. Moreover, the study was cross-sectional, impeding any inference about causality. Another limitation relates to medication use. This was reported by the clinician, but we do not have information about treatment duration, compliance, or whether patients currently not using ADHD medication had previously used such medication. Also, the number of ADHD patients without medication was low, making these data less robust. Some may consider it a limitation not to adjust for other comorbidities in the analyses, such as anxiety and depression. Both ADHD and sleep disorders are often co-morbid with other psychiatric and somatic disorders. We recently reported that the prevalence of anxiety/depression among our adult ADHD patients was 66.3% compared to 16.9% among the controls (*p* < 0.001; Brevik et al., 2017). Having psychiatric co-morbidity will likely add to the general symptom load, and also possibly influence the associations between ADHD and sleep problems. However, in recent years it has been recognized that sleep disorders need independent clinical attention as separate disorders regardless of other conditions (American Academy of Sleep Medicine, 2014). One reason for this shift is that it is often impossible to determine whether the sleep problem is caused by or causing, for instance, depression. Adjusting for other co-morbidities may therefore deflate the importance of the sleep problem. Furthermore, controls were recruited without exclusion criteria. This implies that some controls may have had ADHD or ADHD-like symptoms. This would reduce the chance of finding differences when comparing controls with the ADHD group. According to our IRB approved protocol, our intention was to recruit similar numbers of cases and controls. However, due to the slightly lower response rates from the controls, the final number of valid data collected from controls was lower than from the patient group. Another limitation was that we do not report data about the severity of ADHD. This makes the interpretation of medication use difficult, as untreated patients may suffer from less severe disorder, or patients not on medication may have stopped taking them because of inefficacy.

In conclusion, our study showed that adult ADHD patients reported considerably more of both general sleep impairment and specific sleep-related problems, including self-reported restless legs and cataplexy, compared to a representative control group. Current use of ADHD medication was not associated with worse sleep, but in fact fewer sleep-related symptoms. There were small differences in the sleep variables in relation to the ADHD subtype, except for better sleep quality and less restless legs among patients belonging to the inattentive subtype. Taken together, these findings underline the importance of screening for sleep disorders as part of the diagnostic assessment and treatment of ADHD.

## Author contributions

All authors contributed substantially to the conception/design of the work, or the analysis or interpretation of the data. Furthermore, all authors drafted or revised the paper, and approved the final version, and agreed to be accountable for all aspects of the work.

### Conflict of interest statement

JH has received lecture honoraria as part of continuing medical education programs sponsored by Novartis, Eli Lilly and Company, and Janssen-Cilag. The other authors declare that the research was conducted in the absence of any commercial or financial relationships that could be construed as a potential conflict of interest.

## Appendix

Sleep questionnaire.

Have you ever had sleep problems lasting 1 month or longer?

     Yes      No

Have you ever been prescribed sleep medications?

     Yes      No

How will you rate your sleep?^1^

     very good      good      neither good nor bad      bad      very bad

How much sleep do you on average get per day? ____hours ____minutes

Are you a morning- or evening person?

     definite morning type      moderate morning type      intermediate type      moderate evening type      definite evening type

During the past 4 weeks, how often did you fall asleep unintentionally or had to fight to stay awake during the day?^2^

     never      sometimes      usually      always

During the past 4 weeks, how often did you experience sudden loss of muscle tone (e.g. knees unlocking) by emotional reactions like laughter, anger or fear?^3^

     never      sometimes      usually      always

During the past 4 weeks, do you know (possibly through others) how often you have snored loudly?

     never      sometimes      usually      always

During the past 4 weeks, do you know (possibly through others) how often you have had breathing pauses or stopped breathing in your sleep?

     never      sometimes      usually      always

During the past 4 weeks, how often did you experience restless or crawling feelings in your legs in the evening or at night that went away if you moved your legs?^4^

     never      sometimes      usually      always

During the past 4 weeks, do you know (possibly through others) how often you have had repeated rhythmic leg jerks or leg twitches during your sleep?^5^

     never      sometimes      usually      always

^1^Proxy for sleep quality.

^2^Proxy for excessive daytime sleepiness.

^3^Proxy for cataplexy (sign of narcolepsy).

^4^Proxy for restless legs.

^5^Proxy for periodic limb movements in sleep.
